# Improving Healthcare Workers' Adherence to Surgical Safety Checklist: The Impact of a Short Training

**DOI:** 10.3389/fpubh.2021.732707

**Published:** 2022-02-08

**Authors:** Davide Ferorelli, Marcello Benevento, Luigi Vimercati, Lorenzo Spagnolo, Luigi De Maria, Antonio Caputi, Fiorenza Zotti, Gabriele Mandarelli, Alessandro Dell'Erba, Biagio Solarino

**Affiliations:** ^1^Section of Legal Medicine, Department of Interdisciplinary Medicine, University of Bari, Bari, Italy; ^2^Section of Occupational Medicine, Department of Interdisciplinary Medicine, University of Bari, Bari, Italy; ^3^Section of Clinical Risk Management, Policlinico University Hospital of Bari, Bari, Italy; ^4^Section of Criminology and Forensic Psychiatry, Department of Interdisciplinary Medicine, University of Bari, Bari, Italy

**Keywords:** surgical safety checklist, risk management, clinical governance, safety culture, patient's safety

## Abstract

**Background:**

Although surgery is essential in healthcare, a significant number of patients suffer unfair harm while undergoing surgery. Many of these originate from failures in non-technical aspects, especially communication among operators. A surgical safety checklist is a simple tool that helps to reduce surgical adverse events, but even if it is fast to fill out, its compilation is often neglected by the healthcare workers because of unprepared cultural background. The present study aims to value the efficacy of a free intervention, such as a short training about risk management and safety checklist, to improve checklist adherence.

**Methods:**

In March 2019, the medical and nursing staff of the General Surgical Unit attended a two-lesson theoretical training concerning surgical safety and risk management tools such as the surgical safety checklist. The authors compared the completeness of the surgical checklists after and before the training, considering the same period (2 months) for both groups.

**Result:**

The surgical safety checklists were present in 198 cases (70.97%) before the intervention and 231 cases (96.25%) after that. After the training, the compilation adherence increased for every different type of healthcare worker of the unit (surgeons, nurses, anesthetists, and scrab nurses). Furthermore, a longer hospitalization was associated with a higher surgical checklist adherence by the operators.

**Conclusions:**

The results showed that a free and simple intervention, such as a two-lesson training, significantly stimulated the correct use of the surgical safety checklist. Moreover, the checklist adherence increased even for the operators who did not attend the training, maybe because of the positive influence of the colleagues' positive behaviors. As the results were promising with only two theoretical lessons, much more can be done to build a new safety culture in healthcare.

## Introduction

The operating room is a complex system that involves many professionals with different technical tasks. An incompressible risk for the patient is inherent in any type of surgery (especially in emergency or urgent conditions), hence a significant number of patients suffer from harm while undergoing a surgical procedure (1). Around one in twenty patients are exposed to preventable harm, 10% of which was reported in surgery (2). Furthermore, although surgery is an essential resource for health care, the resources invested in it are often inadequate (3). In such a context of limited funds, a surgical error causing serious harm to the patient poses a medical liability issue as well as an ethical one.

The four most frequent consequences of surgical errors are surgical wound infection, anatomical dehiscence, deep venous thrombosis, and surgical mortality (4). This also means an increase in the length of hospitalization and costs. Many surgical adverse events originate from failures in non-technical aspects such as leadership, situation awareness, decision making, and especially communication and teamwork among operators (5, 6).

Some special incidents within healthcare have been included in a list known as “never events,” which are defined as wholly preventable serious healthcare-related adverse events (7). Regarding surgery, there are three such “never events,” all depending on non-technical aspects: wrong-site surgery, retained foreign object, and incorrect implant (8). Despite their name, the surgical “never events” still happen. Therefore, many attempts to improve communicative aspects of healthcare practice have been described in the literature. For example, in the Policlinico University Hospital of Bari (Italy), Ferorelli et al. tried to standardize the communication in handover by developing a handover checklist model, while in the Columbia University Medical Center of New York City, Nakagawa et al. had positive results by developing a 2-h communication skills training program for general surgery residents (9, 10).

A widely employed tool in error management is the checklist, a systematically arranged list of actions, steps, or objects, that allows the user to ensure that all the listed items are considered (11). In 2004, the WHO launched the program “Safe surgery saves life” to improve the safety of surgical care around the world by defining a core set of safety standards that can be applied in all countries and settings. The program regards the surgical checklist as essential and promotes its adoption worldwide (12, 13). Hynes et al., in 2009, demonstrated that the adoption of the surgical checklist reduced the rates of death and complications among surgical patients (14).

From 2013, in Apulia Region, Italy, the Units of Regional Health Service applied a revised surgical safety checklist (CL). The CL comprises four parts (called “times”: transfer, sign-in, time-out, and sign-out), each containing different sections that should be filled by every professional involved (surgeons, anesthetists, nurses, and scrab nurses). Still today, the CL is not drawn up regularly and completely, despite the simplicity of compilation and the commitment to include it into the medical records. The correct utilization of such instruments partly depends on the safety knowledge and safety attitudes of healthcare workers (15).

The present study aims to measure the impact of the two-lessons training concerning risk management in healthcare. The authors verified the presence and completeness of CL in a third-level hospital after and before such intervention.

## Methods

### Context

In March 2019, the Clinical Risk Board of the Apulia Region, together with the Clinical Risk Unit of a third level university hospital (Policlinico University Hospital of Bari, over 1,000 beds), set up an operative group that involved the medical and nursing staff of the General Surgery Unit, which aimed to promote surgical safety. In April 2019, the members of the operative group attended two theoretical lessons of 1.5 h each about surgical safety and the risk management tools held by an expert of the Clinical Risk Unit.

The General Surgery Unit of the Policlinico University Hospital of Bari (which had more than 1,000 beds) has 2 operating rooms and 25 beds. Ten physicians and 24 nurses worked in the unit.

### Intervention

The program contained the following lessons:

- Ricks and safety in a complex system (first session)
- The risk management in healthcare (first session)
- Incident reporting and no-blame culture (second session)
- Clinical safety checklist: history and achievement of a safety tool (second session).

Every physician and nurse in the unit attended at least one of the lessons but the anesthetists and the scrub nurses did not because they rotate between the different surgery units. The authors valued the completeness of the CL in the General Surgery Unit after and before this intervention by checking the clinical records. The participants did not know about the study.

### Study of the Intervention and Measures

First, the investigator retrospectively analyzed the clinical records of the 318 patients discharged before the operative group was activated (Before-training group: January 1, 2019–March 31, 2019). Then, the investigator collected the clinical records of the 280 patients discharged after the CL training (After-training group: April 1, 2019–June 30, 2019).

The recorded data for each patient were as follows: number of clinical records, date of surgical procedure (if performed), type of procedure, length of hospitalization, presence/absence of CL, and completeness of each different section. The completeness of the sections of CL and the presence/absence of CL were measured to assess the efficacy of the intervention in increasing adherence of operators to CL. The single section completeness was used to draw out the defaulting operator in order to measure the differences. The length of hospitalization and the type of surgery were measured to verify their eventual influence on CL adherence.

Excel was employed for data collection, and the statistical analyses were performed by using R software (Pearson χ^2^ test and Unpaired two-sample Wilcoxon test); *p* < 0.05 was considered significant.

### Ethical Considerations

As no experimentations were conducted and no personal data were collected, according to the local regulation, the study did not require any approval from the Ethical Committee.

## Results

A total amount of 598 clinical records of the General Surgery Unit were analyzed. A total of 519 patients (89.79%) had surgery during their hospitalization, with 279 patients in the before-training group (87.74%) and 240 in the after-training group (85.71%) (*p* = 0.47).

According to the type of surgical procedure, the population was divided as follows: 161 patients (78 before-training and 83 after-training) had an intestinal operation; 117 patients (73 before-training and 44 after-training) had a breast operation; 99 patients (49 before-training and 50 after-training) had an hepato-biliary district operation; 45 patients (26 before-training and 19 after-training) had thyroid surgery; 24 patients (13 before-training and 11 after-training) had an operation for hernia; 22 patients (17 before-training and 5 after-training) had an esophagus or stomach surgery; and 51 patients (23 before-training and 28 after-training) had another type of operation (*p* = 0.06).

The CL was present (completely or partially filled) in 198 procedures (70.97%) in the pre-training group, and 231 (96.25%) in the after-training group (*p* < 0.01) (Table 1).

**Table 1:** Completeness of checklists.

**Checklist**	**Before-training**		**After-training**		***p*-value**
Present	198	70.97%	231	96.25%	<0.01
Absent	81	29.03%	9	3.75%	<0.01
All	279		240		

The CL was wholly filled in 57 cases (20.43%) in the before-training group and 105 (43.75%) in the after-training group (*p* < 0.01), while incompletely filled in 141 cases (50.54%) before the training and 126 cases (52.50%) after the training (*p* < 0.01). Among all the incomplete CL, it was pointed out which was the defaulting operator. The “transfer time” comprises one section for the surgeons and one for the nurses, while the “sign-in time,” the “time-out time,” and “sign-out time” contain one section for each of the surgeons, nurses, anesthetists, and scrub nurses. So, every CL contains 14 sections. All the professionals showed a significant increase in CL adherence (Table 2).

**Table 2:** Incomplete checklist per operator.

**Checklists' section**	**Before-training**		**After-training**		***p*-value**
	**Filled (%)**	**Not Filled (%)**	**Filled (%)**	**Not filled (%)**	
S	556 (50.27)	550 (49.73)	861 (89.69)	99 (10.31)	<0.01
N	714 (63.98)	402 (36.02)	874 (91.04)	86 (8.96)	<0.01
A	455 (54.36)	382 (45.64)	554 (76.94)	166 (23.06)	<0.01
SN	513 (324)	324 (38.71)	604 (83.89)	116 (16.11)	<0.01

*S, surgeon; A, anesthetist; N, nurse; SN, scrab nurse*.

The length of hospitalization of each included patient was measured before and after the training. Both the before-training (median = 3; interquartile range: 2–6) and the after-training (median = 4; interquartile range: 2–7) groups have a similar non-parametric distribution of length of hospitalization (*p* = 0.72). Considering all patients, the hospitalization is significantly longer when the CL is present (Median = 3; interquartile range: 2–7) than when it is absent (median = 2; interquartile range: 2–5) (*p* < 0.01) (Table 3).

**Table 3:** Length of hospitalization (days).

**Group**	**Median (interquartile range)**	***p*-value**
Before-training	3 (2–6)	0.72
After-training	4 (2–7)	
All patients – CL present	3 (2–7)	<0.01
All patients – CL absent	2 (2–5)	

*CL, checklist*.

## Discussion

The present study was aimed to value the efficacy of a simple intervention promoting CL adherence. The authors valued the presence and the completeness of the CL in the clinical records of patients discharged from the General Surgery Unit before (before-training group) and after (after-training group) that operators attended a short training about surgical risk management and the use of CL. According to the results, the CL was often incomplete or absent.

Studying adherence to the CL may help to understand how to improve such risk management tools. According to a Brazilian study, the American Society of Anaesthesia score (ASA score) and the length of hospitalization of each patient may influence CL adherence (16). The ASA score provides a baseline metric for the fitness of a patient before undergoing surgery in addition to predicting mortality (17). Similarly, the length of hospitalization is partially a function of patient and disease factors (18). The before-training and after-training groups have similar numbers and types of surgeries performed and length of hospitalization.

The present study noticed a positive association between the length of hospitalization and CL adherence for both after-training groups and before-training groups. A hypothesis may be that the operators believe that those patients with bad fitness and a high length of hospitalization have an elevated risk of experiencing adverse events, causing them to be more careful about safety tools. Hence, even if every patient requires to be treated in a safe condition, and the CL is quite simple and fast to fill out, as suggested by Weaver et al., the shared (and maybe insufficient) safety culture seems to shape the perception of health operators about “normal” behavior related to patient safety and appears to be change-resistant (19). Therefore, it is essential to ceaselessly carry out proactive clinical risk management in order to achieve a new safety culture in healthcare (20).

After the training, it was observed that there was a sharp increase of present CL (from 70.97 to 96.25%). The wholly filled CL increased from 20.43 to 43.75%, and there was a slight increase of incomplete CL (from 50.50 to 52.50%). Even if those results were promising, similar studies reported even better ones (16, 21).

Among all sections of CL, it was possible to identify which operator was the defaulting one. It was just the surgeons and nurses who attended the lessons, as the anesthetists and scrub nurses were not assigned to the General Surgery Unit as they rotated among the different surgical units. Notwithstanding this, all the professionals showed a significant increase in CL compliance. A hypothesis is that the non-trained operators were influenced by the positive behaviors of their colleagues. It means that a simple intervention such as the short theoretical training stimulated a behavioral change that can may be “self-feed.”

In conclusion, as shown by several authors, CL compliance is essentially a cultural issue (22–24). Educate the healthcare workers is necessary in order to achieve a new safety culture and a more general no-blame culture (25). The increase in CL adherence is indeed demonstrated to have a positive association with the reduction of complications, morbidity, and mortality after surgery (26–29). It also means a reduction in costs for the healthcare system through different mechanisms. A reduction of direct costs of beds and therapies, the decrease of adverse events, and the better quality of documentation in clinical records could ensure savings in medical liability costs (30).

The main limitation of the study is that just a few healthcare workers participated in it. Moreover, other clinical data might be considered as variables. Another limitation is the lack of depth analysis of the specific cultural context. The main strength is that the participants did not know about the study.

The next step will be to involve all the professional figures in the training and find new trainers among the training participants (including nurses and anesthetists) to actively involve the operators in risk management. Any clinical and economic results must be longitudinally monitored over time to be able to attract investments in clinical risk governance. The present study reported a significant increase in CL compliance obtained with a short training. To ensure CL adherence among healthcare operators means to promote a new safety culture and to require both an “external” intervention (such as CL training) and an “internal” one (by actively involving operators as trainers).

## Data Availability Statement

The raw data supporting the conclusions of this article will be made available by the authors, without undue reservation.

## Author Contributions

DF conceptualized the study and wrote the original draft. MB was responsible for data curation and analysis. LD, AC, LS, and FZ collaborated in investigation and data collection. LV and GM lead writing, reviewing, and editing. AD and BS supervised the research project. All authors have read and agreed to the published version of the manuscript.

## Conflict of Interest

The authors declare that the research was conducted in the absence of any commercial or financial relationships that could be construed as a potential conflict of interest.

## Publisher's Note

All claims expressed in this article are solely those of the authors and do not necessarily represent those of their affiliated organizations, or those of the publisher, the editors and the reviewers. Any product that may be evaluated in this article, or claim that may be made by its manufacturer, is not guaranteed or endorsed by the publisher.

## Abbreviations

CL: Surgical safety checklist.

## References

[1] Duclos A Chollet F Pascal L Ormando H Carty MJ Polazzi S. Effect of monitoring surgical outcomes using control charts to reduce major adverse events in patients: cluster randomised trial. BMJ. (2020) 371:1–10. 10.1136/bmj.m3840 33148601 PMC7610189

[2] Panagioti M Khan K Keers RN Abuzour A Phipps D Kontopantelis E. Prevalence, severity, and nature of preventable patient harm across medical care settings: systematic review and meta-analysis. BMJ. (2019) 366:l4185. 10.1136/bmj.l4185 31315828 PMC6939648

[3] Funk LM Weiser TG Berry WR Lipsitz SR Merry AF Enright AC. Global operating theatre distribution and pulse oximetry supply: an estimation from reported data. Lancet. (2010) 376:1055–61. 10.1016/S0140-6736(10)60392-3 20598365

[4] Ferorelli D Zotti F Tafuri S Pezzolla A Dalfino L Brienza N. Good medical practices in the use of antibiotic prophylaxis in a surgery ward: results of a 2013 Apulian study. Am J Infect Control. (2015) 43:e79–81. 10.1016/j.ajic.2015.07.021 26315057

[5] Yule S Flin R Paterson-Brown S Maran N. Non-technical skills for surgeons in the operating room: a review of the literature. Surgery. (2006) 139:140–9. 10.1016/j.surg.2005.06.017 16455321

[6] Klaas S Kara M Nikki M Rhona F Simon PB. A ward-round non-technical skills for surgery (WANTSS) taxonomy. J Surg Educ. (2020) 77:369–79. 10.1016/j.jsurg.2019.09.011 31591044

[7] Wahid NNA Moppett SH Moppett IK. Quality of quality accounts: transparency of public reporting of never events in England. A semi-quantitative and qualitative review. J R Soc Med. (2016) 109:190–9. 10.1177/0141076816636367 26933157 PMC4872205

[8] Moppett IK Moppett SH. Surgical caseload and the risk of surgical never events in England. Anaesthesia. (2016) 71:17–30. 10.1111/anae.13290 26597516

[9] Ferorelli D Giandola T Laterza M Solarino B Pezzolla A Zotti F. Handover checklist: Testing a standardization process in an Italian hospital. Risk Manag Healthc Policy. (2017) 10:87–93. 10.2147/RMHP.S129652 28579841 PMC5449138

[10] Nakagawa S Fischkoff K Berlin A Arnell TD Blinderman CD. Communication skills training for general surgery residents. J Surg Educ. (2019) 76:1223–30. 10.1016/j.jsurg.2019.04.001 31005480

[11] Hales BM Pronovost PJ. The checklist-a tool for error management and performance improvement. J Crit Care. (2006) 21:231–5. 10.1016/j.jcrc.2006.06.002 16990087

[12] World Health Organization. WHO Guidelines for Safe Surgery 2009. Geneva: World Health Organization (2009).

[13] Urbach DR Govindarajan A Saskin R Wilton AS Baxter NN. Introduction of surgical safety checklists in Ontario, Canada. N Engl J Med. (2014) 370:1029–38. 10.1056/NEJMsa1308261 24620866

[14] Haynes AB Weiser TG Berry WR Lipsitz SR Breizat A-HS Dellinger EP. A surgical safety checklist to reduce morbidity and mortality in a global population. N Engl J Med. (2009) 360:491–9. 10.1056/NEJMsa0810119 19144931

[15] Ginsburg LR Dhingra-Kumar N Donaldson LJ. What stage are low-income and middle-income countries (LMICs) at with patient safety curriculum implementation and what are the barriers to implementation? A two-stage cross-sectional study. BMJ Open. (2017) 7:e016110. 10.1136/bmjopen-2017-016110 28619782 PMC5726095

[16] Ribeiro L Fernandes GC de Souza EG Souto LC Dos Santos ASP Bastos RR. Safe surgery checklist: filling adherence, inconsistencies, and challenges. Rev Col Bras Cir. (2019) 46:1–12. 10.1590/0100-6991e-20192311 31859726

[17] Helkin A Jain S V. Gruessner A Fleming M Kohman L Costanza M. Impact of ASA score misclassification on NSQIP predicted mortality: a retrospective analysis. Perioper Med. (2017) 6:1–6. 10.1186/s13741-017-0076-1 29238570 PMC5725975

[18] Abela L Pace A Buttigieg SC. What affects length of hospital stay? A case study from Malta. J Health Organ Manag. (2019) 33:714–36. 10.1108/JHOM-10-2018-0280 31625819

[19] Weaver S Lubomksi L Wilson R Pfoh E Martinez K Dy S. Supplement promoting a culture of safety as a patient safety strategy. Ann Intern Med. (2013) 158:369–74. 10.7326/0003-4819-158-5-201303051-00002 23460092 PMC4710092

[20] Euteneier A. Culture of safety and clinical risk management. Unfallchirurg. (2020) 123:22–8. 10.1007/s00113-019-00740-2 31728555

[21] White MC Randall K Capo-Chichi NFE Sodogas F Quenum S Wright K. Implementation and evaluation of nationwide scale-up of the surgical safety checklist. Br J Surg. (2019) 106:e91–102. 10.1002/bjs.11034 30620076 PMC6519364

[22] Munn Z Giles K Aromataris E Deakin A Schultz T Mandel C. Mixed methods study on the use of and attitudes towards safety checklists in interventional radiology. J Med Imaging Radiat Oncol. (2018) 62:32–8. 10.1111/1754-9485.12633 28600853

[23] Treadwell JR Lucas S Tsou AY. Surgical checklists: a systematic review of impacts and implementation. BMJ Qual Saf. (2014) 23:299–318. 10.1136/bmjqs-2012-001797 23922403 PMC3963558

[24] Fudickar A Hörle K Wiltfang J Bein B. The effect of the WHO surgical safety checklist on complication rate and communication. Dtsch Arztebl Int. (2012) 109:695–701. 10.3238/arztebl.2012.0695 23264813 PMC3489074

[25] Rodziewicz TL Houseman B Hipskind JE. Medical Error Reduction and Prevention. Treasure Island, FL: StatPearls Publishing (2021). 29763131

[26] Haugen AS Søfteland E Almeland SK Sevdalis N Vonen B Eide GE. Effect of the World Health Organization checklist on patient outcomes: a stepped wedge cluster randomized controlled trial. Ann Surg. (2015) 261:821–8. 10.1097/SLA.0000000000000716 24824415

[27] van Klei WA Hoff RG van Aarnhem EEHL Simmermacher RKJ Regli LPE Kappen TH. Effects of the introduction of the WHO “surgical safety checklist” on in-hospital mortality: a cohort study. Ann Surg. (2012) 255:44–9. 10.1097/SLA.0b013e31823779ae 22123159

[28] Mayer EK Sevdalis N Rout S Caris J Russ S Mansell J. Surgical checklist implementation project: the impact of variable WHO checklist compliance on risk-adjusted clinical outcomes after national implementation: a longitudinal study. Ann Surg. (2016) 263:58–63. 10.1097/SLA.0000000000001185 25775063

[29] de Jager E Gunnarsson R Ho YH. Implementation of the World Health Organization surgical safety checklist correlates with reduced surgical mortality and length of hospital admission in a high-income country. World J Surg. (2019) 43:117–24. 10.1007/s00268-018-4703-x 29947986

[30] Abbott RL Weber P Kelley B. Medical professional liability insurance and its relation to medical error and healthcare risk management for the practicing physician. Am J Ophthalmol. (2005) 140:1106–11. 10.1016/j.ajo.2005.07.020 16376659

