# Supplementary material for: Improving Healthcare Workers' Adherence to Surgical Safety Checklist: The Impact of a Short Training
Source: Front Public Health. 2022 Feb 8;9:732707. doi: 10.3389/fpubh.2021.732707 (PMC8860967; doi:10.3389/fpubh.2021.732707)
Supplement: Supplementary file 1 [file Data_Sheet_1.docx]

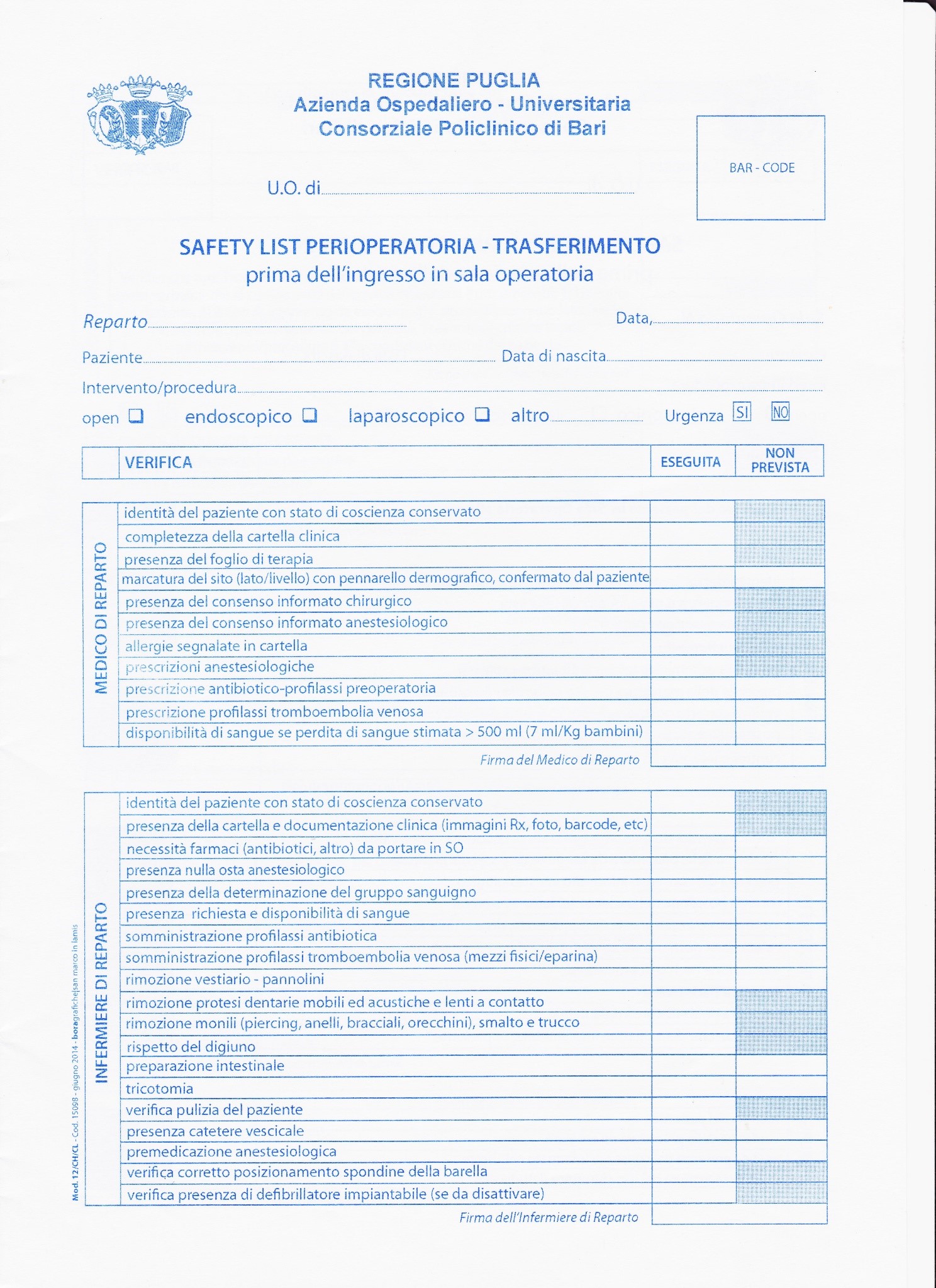


Figure 1: Safety Checklist - Transfer


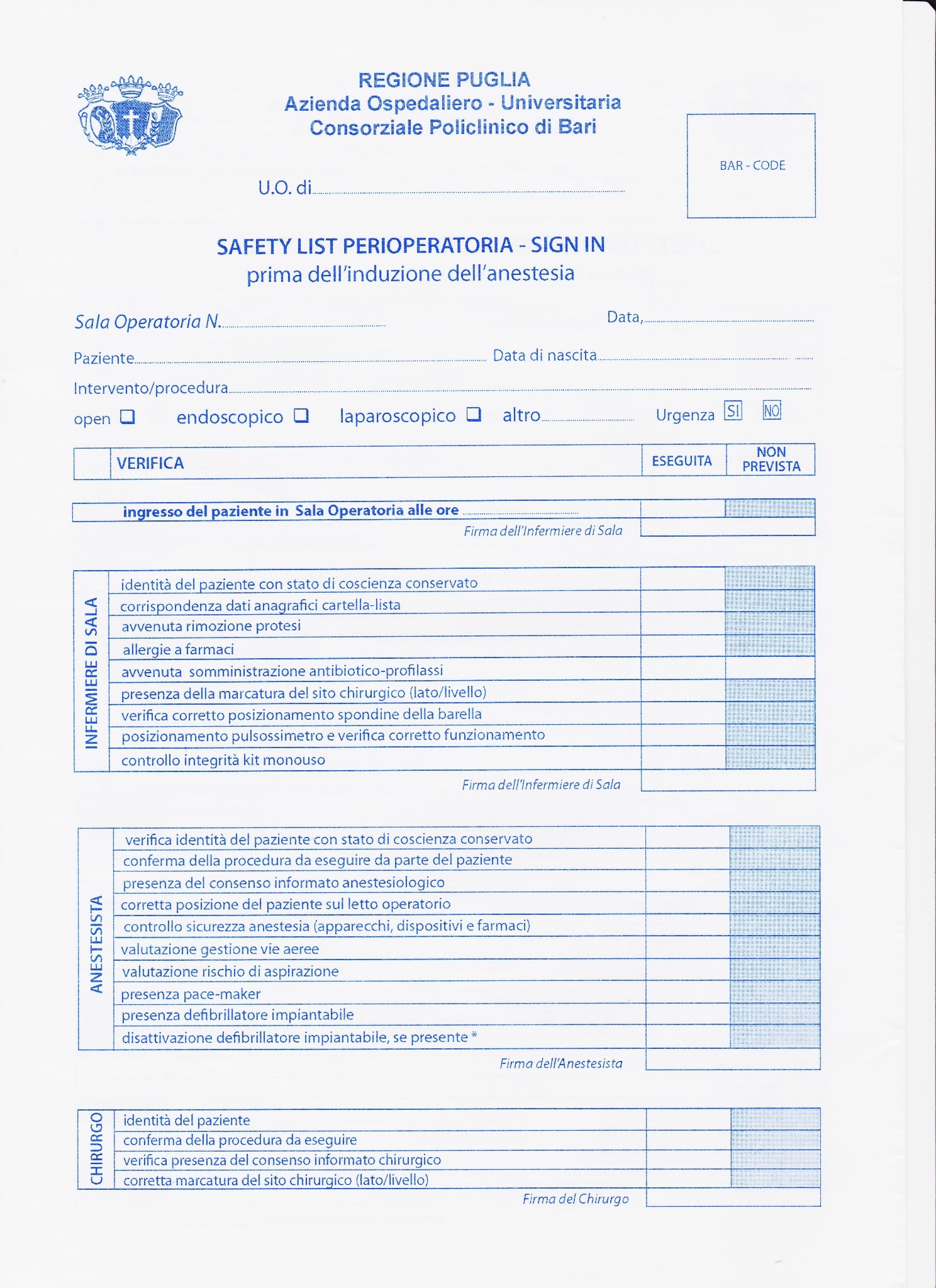


Figure 2: Safety Checklist - Sign-in


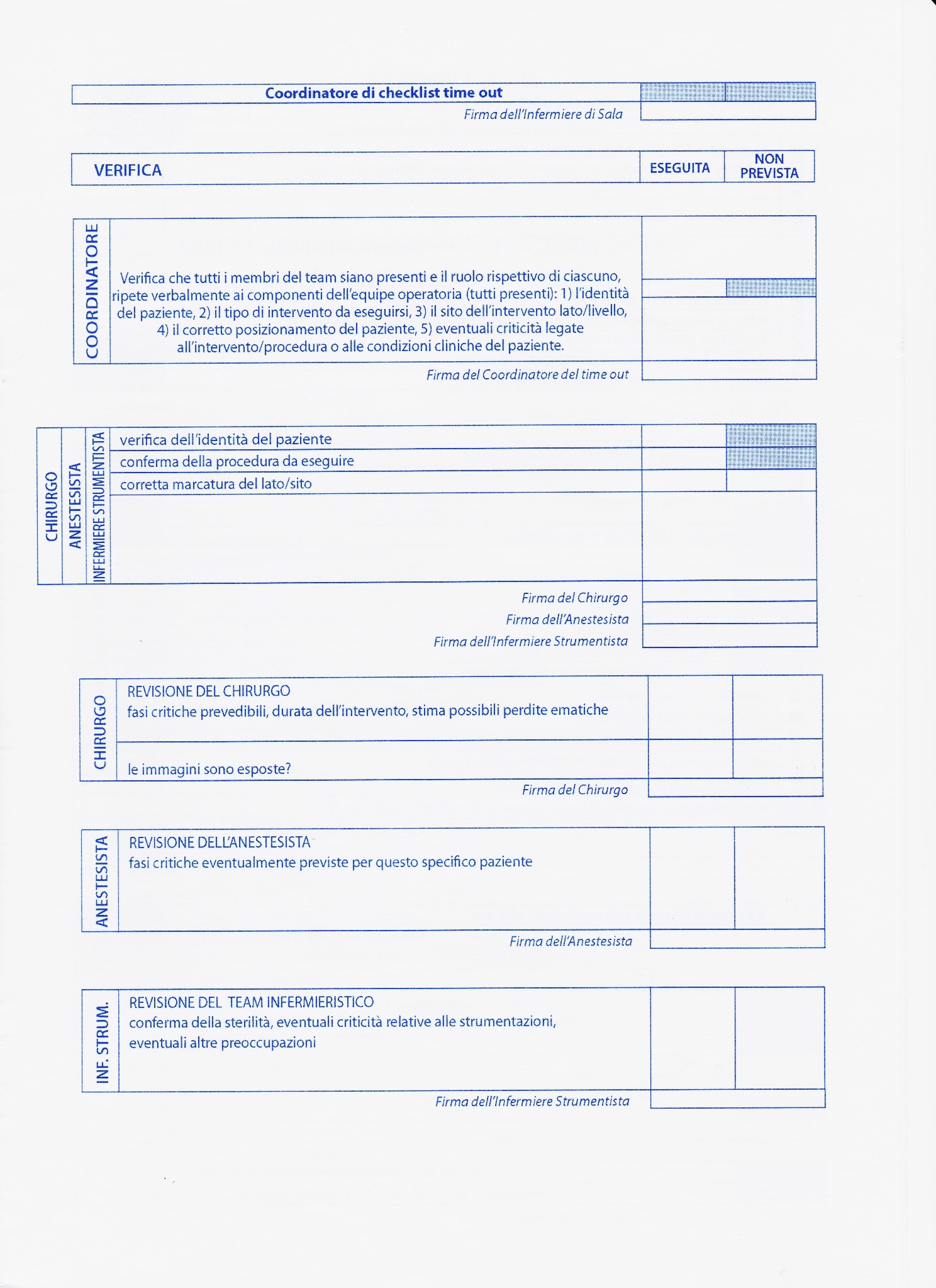


Figure 3: Safety Checklist - Time-out


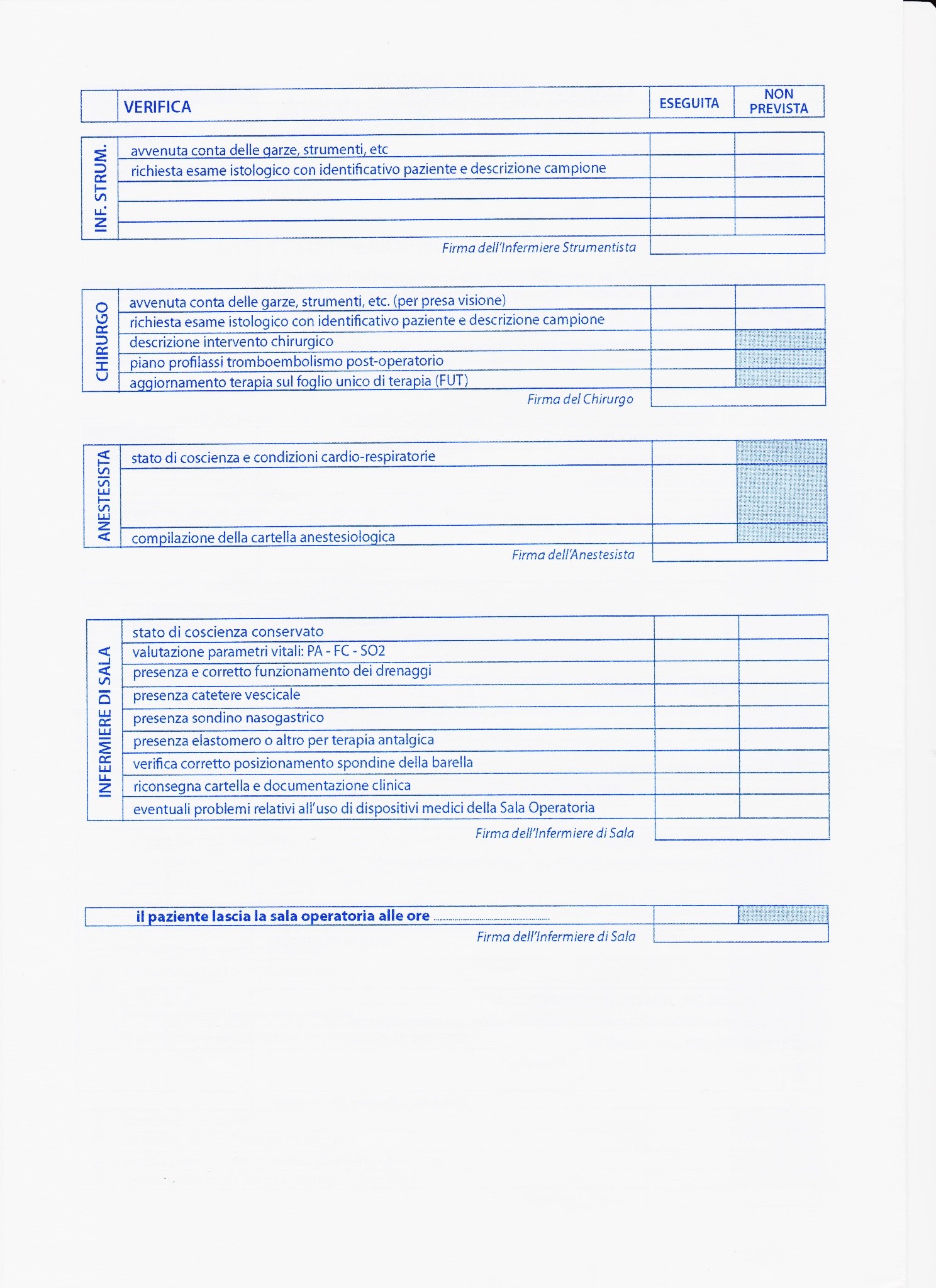


Figure 4: Safety Checklist - Sign-out
